# Screening and Validation of Appropriate Reference Genes for Real-Time Quantitative PCR under PEG, NaCl and ZnSO_4_ Treatments in *Broussonetia papyrifera*

**DOI:** 10.3390/ijms242015087

**Published:** 2023-10-11

**Authors:** Mengdi Chen, Zhengbo Wang, Ziyuan Hao, Hongying Li, Qi Feng, Xue Yang, Xiaojiao Han, Xiping Zhao

**Affiliations:** 1 College of Horticulture and Plant Protection, Henan University of Science and Technology, Luoyang 471023, China; a1442388073a@163.com (M.C.);; 2 Key Laboratory of Tree Breeding of Zhejiang Province, Research Institute of Subtropical Forestry, Chinese Academy of Forestry, Hangzhou 311400, China

**Keywords:** real-time quantitative PCR, internal reference genes, *Broussonetia papyrifera*, abiotic stresses, gene expression

## Abstract

Real-time quantitative PCR (RT-qPCR) has a high sensitivity and strong specificity, and is widely used in the analysis of gene expression. Selecting appropriate internal reference genes is the key to accurately analyzing the expression changes of target genes by RT-qPCR. To find out the most suitable internal reference genes for studying the gene expression in *Broussonetia papyrifera* under abiotic stresses (including drought, salt, and ZnSO_4_ treatments), seven different tissues of *B. papyrifera*, as well as the roots, stems, and leaves of *B. papyrifera* under the abiotic stresses were used as test materials, and 15 candidate internal reference genes were screened based on the transcriptome data via RT-qPCR. Then, the expression stability of the candidate genes was comprehensively evaluated through the software geNorm (v3.5), NormFinder (v0.953), BestKeeper (v1.0), and RefFinder. The best internal reference genes and their combinations were screened out according to the analysis results. *rRNA* and *Actin* were the best reference genes under drought stress. Under salt stress, *DOUB*, *HSP*, *NADH*, and *rRNA* were the most stable reference genes. Under heavy metal stress, *HSP* and *NADH* were the most suitable reference genes. *EIF3* and *Actin* were the most suitable internal reference genes in the different tissues of *B. papyrifera*. In addition, *HSP*, *rRNA*, *NADH*, and *UBC* were the most suitable internal reference genes for the abiotic stresses and the different tissues of *B. papyrifera*. The expression patterns of *DREB* and *POD* were analyzed by using the selected stable and unstable reference genes. This further verified the reliability of the screened internal reference genes. This study lays the foundation for the functional analysis and regulatory mechanism research of genes in *B. papyrifera*.

## 1. Introduction

*Broussonetia papyrifera* is a deciduous tree of the genus Broussonetia in the Moraceae family. It is distributed in most parts of China and Southeast Asia. It is a typical native tree species and a pioneer plant [1]. *B. papyrifera* has the advantages of easy reproduction, strong stress resistance, and fast growth, and is widely used in the fields of feed, papermaking, and vegetation restoration [2,3]. Moreover, *B. papyrifera* has medicinal values, as well as flavonoids, polyphenols, and fructose contents that are much higher than those of other plants [4]. Flavonoid derivatives in *B. papyrifera* have inhibitory effects on cancer cells [5], and polyphenols can inhibit coronavirus proteases [6]. Generally speaking, *B. papyrifera* is a woody plant with great potential for development, combining economic value and excellent resistance. At the same time, due to the characteristics of its growing environment, the *B. papyrifera* also has a strong ability to tolerate a variety of unfavorable environments, such as drought, salt, and heavy metals [7,8,9]. Currently, research on *B. papyrifera* focuses on their breeding [10], physiological characteristics [11], medicinal [12], and pasture values [13], but less research has been performed on the molecular mechanisms of their stress tolerance. As a pioneer tree species widely cultivated in harsh environments, stress resistance is a hotspot in molecular biology research in *B. papyrifera* [14]. Therefore, it is important to carry out research on the molecular mechanism of *B. papyrifera* for resistance breeding and the genetic improvement of *B. papyrifera*.

Real-time quantitative PCR (RT-qPCR) has many advantages, such as convenience, strong specificity, and high sensitivity, and is an effective means to study gene functions [15]. However, the accuracy of RT-qPCR is affected by various factors, such as RNA integrity, cDNA quality, sample dilution factor, and experimental operations [16]. In the study of the expression levels and regulation mechanisms of plant functional genes, the optimization of internal reference genes is the key and the basis for correcting and normalizing the expression of the functional genes [17]. Therefore, the introduction of appropriate internal reference genes is crucial for the normalized analysis of target gene expression [18]. An ideal internal reference gene should be the gene that can be stably expressed in cells and whose expression level is almost not disturbed by the external environment. It is generally the housekeeping gene that maintains the basic life activities of cells, such as *Actin*, *18s Ribosome RNA*, *Tublin* and *Ubiquitin*, etc. [19,20]. However, many studies have proved that the transcription levels of the housekeeping genes may change with different species, tissues, and organs [21,22,23]. Therefore, appropriate internal reference genes should be selected according to specific experimental conditions to reduce experimental errors. However, there has been no report on the screening of the internal reference genes in *B. papyrifera*. This limits the research on the regulation mechanisms of gene expression in *B. papyrifera* under adversity stresses.

In this study, 15 candidate internal reference genes (*NADH*, *L13*, *EIF3*, *HIS*, *Actin*, *PP2A*, *DOUB*, *UBE2*, *UBC*, *PTB*, *rRNA*, *GAPDH*, *HSP*, *RPL8*, and *TUA*) were selected based on the transcriptome data in *B. papyrifera*. The RT-qPCR technology, and the geNorm (Version 3.5) [24], NormFinder (version 0.953) [25], and BestKeeper (version 1.0) [26] software were used to analyze the expression stability of the candidate internal reference genes under the abiotic stresses (i.e., drought stress, salt stress, and heavy metal stress) and in different tissues. In addition, the online analysis tool RefFinder [27] was used to comprehensively evaluate the results obtained by the above software. Then, the selected internal reference genes were verified with target genes *DREB* and *POD*. This study is the first to screen and verify the internal reference genes used for RT-qPCR normalization in *B. papyrifera* under the abiotic stresses and the different tissues, which lays the foundation for gene expression analysis in *B. papyrifera*.

## 2. Results

### 2.1. Determination of Primer Specificity and Amplification Efficiency

**Figure 1:**
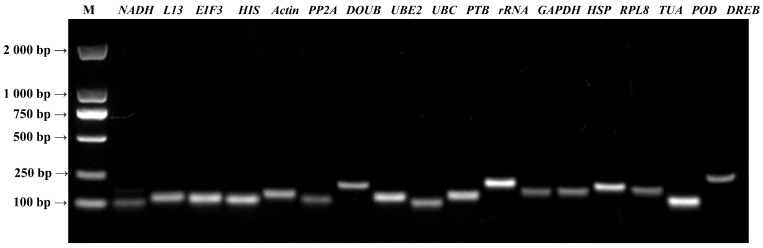
Agarose gel electrophoresis of the conventional PCR products of the candidate internal reference genes in *B. papyrifera*.

**Figure 2:**
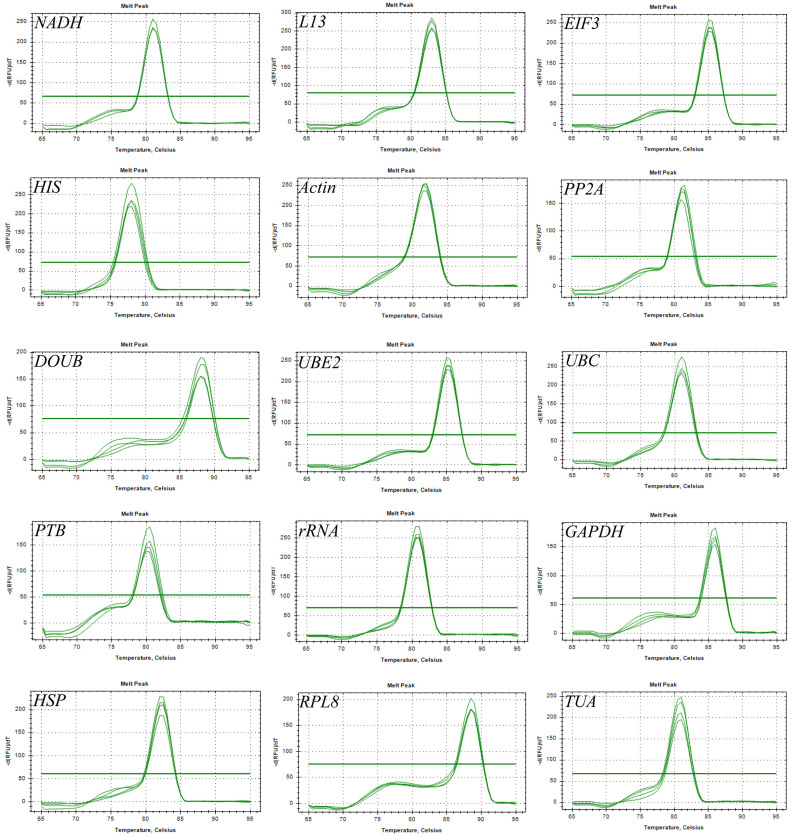
Melting curves of the candidate reference genes in *B. papyrifera*.

The PCR amplifications were performed using equal amounts of the mixed cDNA as templates (Figure 1). The target fragments were unique and bright for all the primers, without primer dimers and non-specific amplifications. The band sizes were in line with the expected values. The primer specificity was verified by the RT-qPCR technology (Figure 2). The melting curves of individual genes showed a single melting peak. This indicates that those primers can perform specific amplifications. After calculation, the amplification efficiency of each candidate internal reference gene was between 90.26–117.99%, and the correlation coefficient (*R*^2^) was between 0.987–0.999 (Table 1). Therefore, those primers achieved good specificity and efficiency for amplifying the candidate genes, suggesting that the candidate genes can be used in subsequent experiments.

### 2.2. C_t_ Values of the Internal Reference Genes

**Figure 3:**
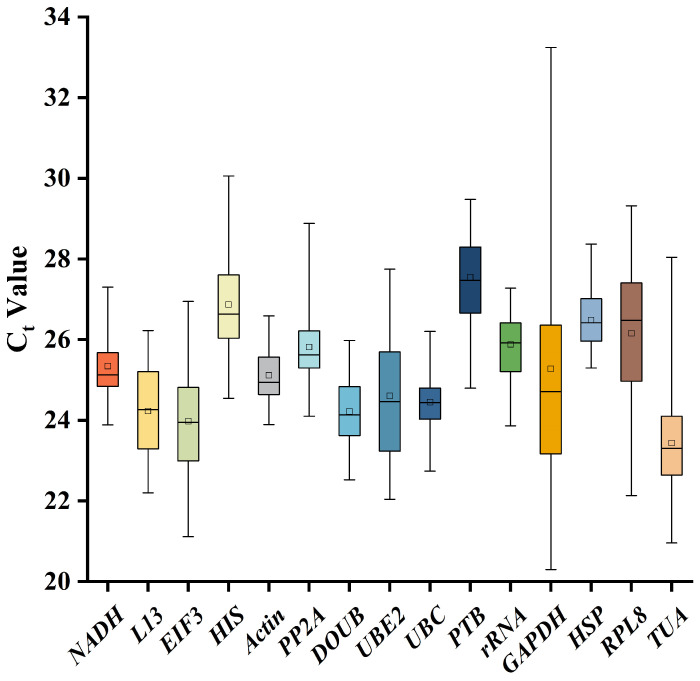
Box plot of the *C*_t_ values of the 15 candidate reference genes in *B. papyrifera.* The box represents the concentrated range of *C*_t_ values. The horizontal line in the middle of the box represents the median value, and the black square represents the average value. The upper and lower edges of the box represent the upper and lower quartiles, respectively. The upper and lower ends of the box represent the maximum and minimum values of the gene, respectively.

The cycle threshold *(C*_t_) value is inversely proportional to the gene expression level. A low *C*_t_ value reflects a high gene expression level. In the analysis of the box plot (Figure 3), the *C*_t_ values of most of the genes were between 22 and 28, indicating moderate expression abundances. As it can be seen in the box plot, the *GAPDH* gene has a broad range of *C*_t_ values (20.30–33.24), indicating a low gene expression stability. However, the *Actin* gene has a narrow range of *C*_t_ values (23.90–26.59), indicating a high gene expression stability. Therefore, different internal reference genes have different expression levels under the abiotic stresses and in the different tissues of *B. papyrifera*. According to the range of *C*_t_ values, the expression level of the *Actin* gene was stable. Therefore, the *Actin* gene was the best candidate internal reference gene.

### 2.3. geNorm Analysis

**Figure 4:**
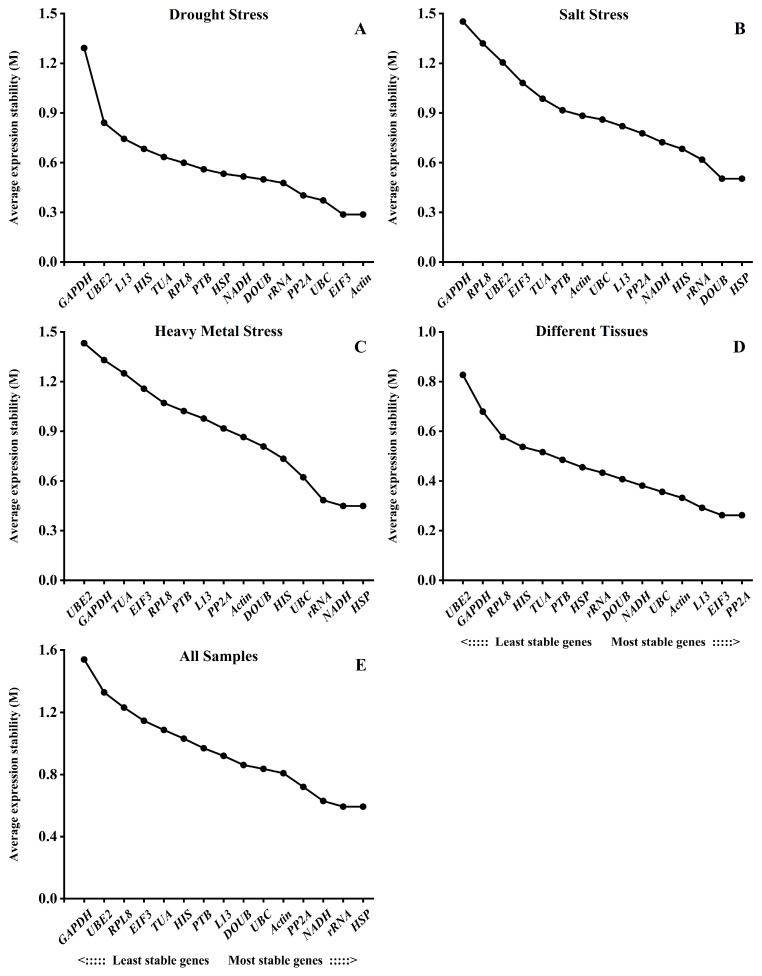
Average expression stability of the 15 candidate reference genes in *B. papyrifera* analyzed by the geNorm program. (**A**) Drought stress, (**B**) salt stress, (**C**) heavy metal stress, (**D**) different tissues, (**E**) all samples.

The M value of the expression stability of each candidate internal reference gene under the drought stress and the different tissues was calculated by the geNorm program. The program takes M = 1.5 as the critical value. The smaller the value of M, the more stable the internal reference gene [24]. The geNorm analyses are shown in Figure 4. The M values of each candidate internal reference gene under the abiotic stresses and in the different tissues were lower than 1.5, suggesting that the expression level of each candidate internal reference gene was stable. The expression levels of the *Actin* and *EIF3* were stable under the drought stress. The *DOUB* and *HSP* were the most stable reference genes under the salt stress. The expression levels of the *NADH* and *HSP* were the most stable under the heavy metal stress. However, among the different tissues of *B. papyrifera*, the *PP2A* and *EIF3* were the most stable genes among the 15 reference genes. Comprehensive analysis of the expression levels of the 15 candidate internal reference genes in all the samples showed that the M values of the other genes were less than 1.5, except for the *GAPDH,* whose M values were greater than 1.5. The order of expression stability from high to low is *HSP* = *rRNA* > *NADH* > *PP2A* > *Actin* > *UBC* > *DOUB* > *L13* > *PTB* > *HIS* > *TUA* > *EIF3* > *RPL8* > *UBE2* > *GAPDH*. Therefore, the *HSP* and *rRNA* genes are the most stable internal reference genes in all the samples, and the *GAPDH* was the least stable one.

**Figure 5:**
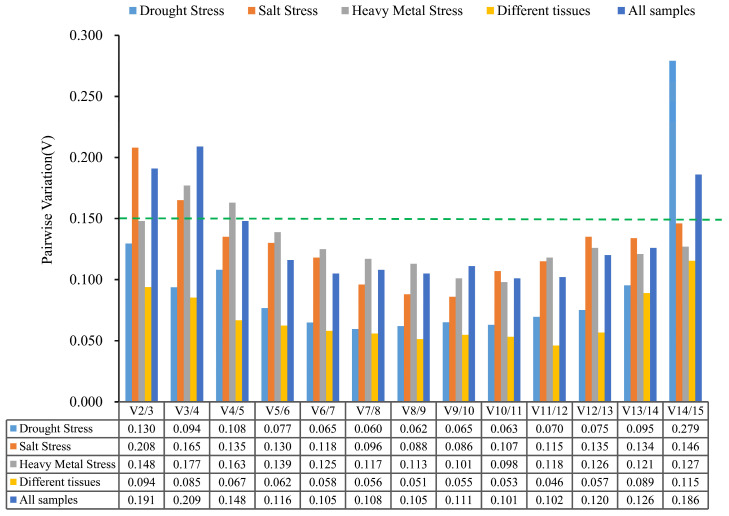
Analysis of the paired variation of the 15 candidate internal reference genes in *B. papyrifera* by geNorm program. The optimal number of candidate reference genes required for accurate normalization was determined by paired variation V_n/(n+1)_. The threshold value of V_n/(n+1)_ was 0.15. When V_n/(n+1)_ is less than 0.15, n genes can ensure stable and reliable results.

Determining the optimal number of internal reference genes can reduce the bias and fluctuation caused by a single internal reference gene. In Figure 5, the V_2/3_ of *B. papyrifera* were 0.130, 0148, and 0.094 under the drought stress, under the heavy metal stress, and in the different tissues, respectively, all of which were less than 0.15. This shows that the results of selecting two internal reference genes were stable and reliable, and thus there was no need to select more than two reference genes. Under the salt stress and all samples, the coefficients of variation were V_2/3_ (0.208) and V_2/3_ (0.191), both of which were higher than the critical value 0.15. Both V_4/5_ (0.135) and V_4/5_ (0.148) were less than 0.15. This indicates that the samples under the salt stress and all samples need to introduce four internal reference genes for correction to keep the results stable and reliable.

### 2.4. NormFinder Analysis

NormFinder needs to convert the *C*_t_ values of the genes into relative expressions before analyzing the data. Then, the stability of the candidate internal reference genes was sorted based on variance analysis. A smaller expression stability value of the candidate internal reference gene indicates a more stable expression [25]. The NormFinder analysis results are shown in Table 2. Under the drought stress and the salt stress, the most stable internal reference gene was the *DOUB*, and the least stable gene was the *GAPDH*. Under the heavy metal stress, the most stable internal reference gene was the *HSP*. In the different tissues, the *rRNA* has a relatively stable expression. The ranking of the gene expression stability in all samples from high to low was: *rRNA* (0.338) > *HSP* (0.383) > *NADH* (0.495) > *PP2A* (0.691) > *UBC* (0.738) > *Actin* (0.751) > *DOUB* (0.753) > *PTB* (0.792) > *HIS* (0.966) > *L13* (1.028) > *TUA* (1.090) > *EIF3* (1.277) > *RPL8* (1.598) > *UBE2* (1.652) > *GAPDH* (2.786). Therefore, the *rRNA* (0.338) was the most stable gene in all samples, and the *GAPDH* (2.786) was the least stable gene.

### 2.5. BestKeeper Analysis

The Bestkeeper mainly evaluates the stability of genes by comparing the SD values among *C*_t_ values of the candidate internal reference genes [26]. The analysis results of the Bestkeeper are shown in Table 3. Under the drought stress, the *UBC* was the most stable reference gene, and the *GAPDH* was the least stable gene. Under the salt stress, the *HSP* was the most stable reference gene, and the *GAPDH* was the least stable gene. Under the heavy metal stress, the *UBC* was the most stable reference gene, and the *UBE2* was the least stable gene. In addition, the *HSP* was the most stable reference gene, and the *UBE2* was the least stable gene in the different tissues. In all samples, the ranking of the gene expression stability from high to low was: *HSP* (0.518) > *UBC* (0.535) > *NADH* (0.607) > *DOUB* (0.615) > *Actin* (0.643) > *PP2A* (0.647) > *rRNA* (0.672) > *TUA* (0.886) > *L13* (0.913) > *PTB* (0.917) > *HIS* (1.018) > *EIF3* (1.023) > *RPL8* (1.353) > *UBE2* (1.477) > *GAPDH* (2.123). It indicates that the *HSP* (0.518) was the most stable gene in all samples, and the *GAPDH* (2.123) was the least stable gene.

### 2.6. RefFinder Analysis

To avoid the error caused by the evaluation program of a single internal reference gene, the online analysis tool RefFinder was used to calculate the geometric mean of the gene expression stability rankings of the above three programs (geNorm, NormFinder, and Bestkeeper). The smaller the geometric mean, the more stable the gene expression [27]. From Table 4, under the drought stress, the *rRNA* and *Actin* were the most stable internal reference genes, and the *GAPDH* was the least stable gene. Under the salt stress, the *DOUB* and *HSP* were the most stable internal reference genes, and the *GAPDH* was the least stable gene. Under the heavy metal stress, the *HSP* and *NADH* were the most stable internal reference genes, and the *UBE2* was the least stable gene. In the different tissues, the *EIF3* and *Actin* were the most stable internal reference genes, and the *UBE2* was the least stable gene. The ranking of the gene expression stability in all samples was: *HSP* (1.414) > *rRNA* (1.627) > *NADH* (3) > *UBC* (3.761) > *PP2A* (5.091) > *Actin* (5.477) > *DOUB* (5.856) > *PTB* (8.459) > *L13* (9.24) > *HIS* (9.975) > *TUA* (10.158) > *EIF3* (12) > *RPL8* (13) > *UBE2* (14) > *GAPDH* (15). Among them, the *HSP* and *rRNA* were identified as the most stable internal reference genes in all samples, and the *GAPDH* was the least stable gene in all samples.

### 2.7. Verification of the Expression Stability of the Internal Reference Genes

**Figure 6:**
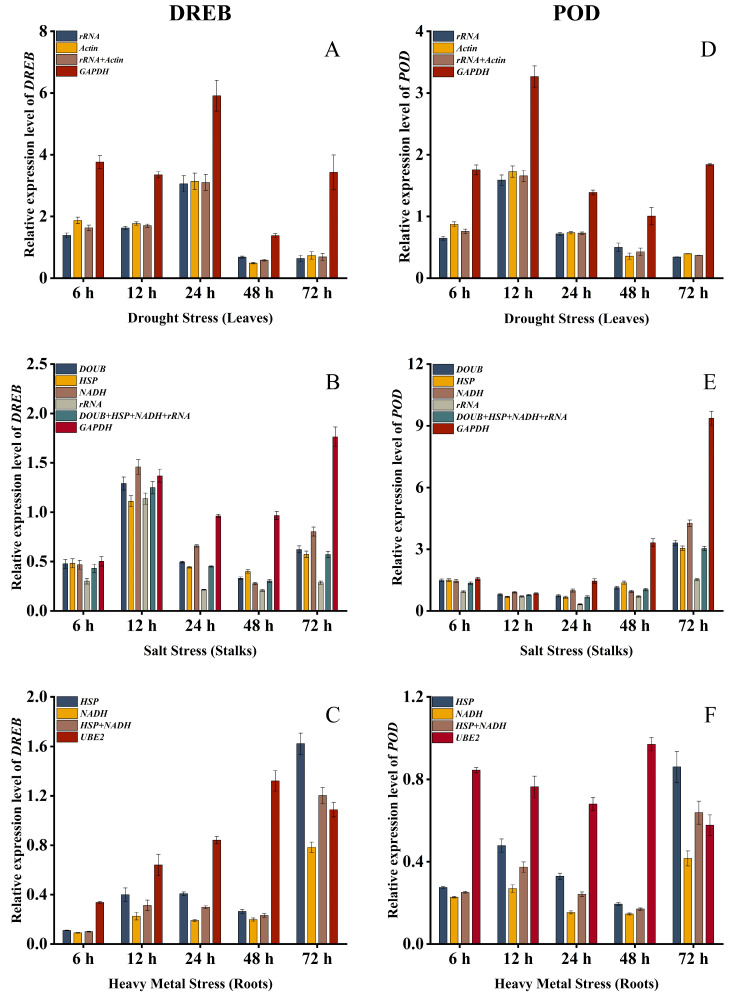
Using *DREB* and *POD* to verify the expression stability of internal reference genes screened under the abiotic stress in *B. papyrifera.* (**A**,**D**): Drought stress (leaves); (**B**,**E**): Salt stress (stalks); (**C**,**F**): Heavy metal stress (roots). The error bars indicate the standard deviations (SDs).

To verify the reliability of the selected internal reference genes, the most stable and least stable internal reference genes screened by the ReFinder program were used as normalization factors. Then, the *DREB* and *POD* gene expression levels were independently validated. As shown in Figure 6, there were great differences in the *DREB* and *POD* expression levels obtained by using different internal reference genes. When the selected stable genes were used alone or in combination as normalized internal reference genes, the relative expression patterns of the *DREB* and *POD* genes showed a similar trend. On the contrary, when relatively unstable genes were used for relative quantification, the relative expression levels of the *DREB* and *POD* were quite different.

Under the drought stress, when the most stable internal reference genes *rRNA*, *Actin,* and their combination (*rRNA* + *Actin*) were used, the expression levels of the *DREB* and *POD* generally showed a trend of increasing first and then decreasing. The *DREB* and *POD* expression level reached the peak at 24 h, and 12 h, respectively. However, when the least stable gene *GAPDH* was used for calculation, the expression levels of the *DREB* and *POD* showed an overall trend of first increasing, then decreasing, followed by increasing. Under the salt stress, when the most stable internal reference genes *DOUB*, *HSP*, *NADH*, *rRNA,* and their combination (*DOUB* + *HSP* + *NADH* + *rRNA*) were used as candidate internal reference genes, the relative expression of the *DREB* showed an overall trend of first increasing, and reached the peak at 12 h. The relative expression of the *POD* decreased first and then increased, and reached a peak at 72 h, with its expression remaining at a low level. When calculated with the unstable gene *GAPDH*, the *DREB* reached the peak at 72 h. Although the *POD* gene expression also reached its peak at 72 h, its expression remained at a high level. Under the heavy metal stress, when the stable internal reference genes *HSP*, *NADH,* and their combination (*HSP* + *NADH*) were used, the relative expression levels of the *DREB* and *POD* both reached their peaks at 72 h. When using the least stable internal reference gene *UBE2*, the *DREB* and *POD* reached their peaks at 48 h. Therefore, the selection of internal reference genes has a great impact on the expression levels of the target genes. Appropriate internal reference genes are conducive to obtaining accurate RT-qPCR results. Using unstable reference genes can lead to unreliable results.

## 3. Discussion

With the development of molecular biology research, the study of key genes controlling plant stress tolerance and its molecular stress tolerance mechanism will provide important information for plant breeding [28]. The RT-qPCR is one of the main methods for analyzing gene expression levels and regulatory patterns [29]. Selection of internal reference genes with stable expression levels is the key to accurately analyzing the target gene expression [30]. The screening of the internal reference genes in combination with the plant transcriptome database is one of the most effective methods for the research in non-model plants [31]. It has been applied in various plants such as *Malpighia emarginata* [32], *Sinocalycanthus chinensisb* [33], and *Oryza sativab* [34]. Therefore, through the transcriptome database, this study screened a batch of stable candidate internal reference genes, *NADH*, *L13*, *EIF3*, *HIS*, *Actin*, *PP2A*, *DOUB*, *UBE2*, *UBC*, *PTB*, *rRNA*, *GAPDH*, *HSP*, *RPL8*, and *TUA*. Then, their expression stability under abiotic stresses (i.e., drought stress, salt stress, and heavy metal stress) and in seven different tissues were studied.

Due to the differences in the operational logic and statistical methods used in each program, the ranking of the expression stability of the internal reference genes were slightly different among the three programs. For example, under the drought stress, the *Actin* and *EIF3* genes are the most suitable reference genes verified by geNorm software. The *DOUB* and *rRNA* genes are the most stable internal reference genes analyzed by the Normfinder software. The *UBC* and *rRNA* genes are the most stable reference genes verified by the Bestkeeper. This phenomenon also appeared in *Carya illinoinensis* [35], *Passiflora edulis* [36], *Forsythia suspensa* [37], etc. Therefore, in order to avoid the one-sidedness of the analysis caused by a single piece of software, scholars usually choose the RefFinder as the comprehensive analysis program for internal reference gene stability. It is widely used in reference gene screening studies [38,39]. In this study, RefFinder was used to comprehensively evaluate the results of the above three kinds of software to determine the ranking of the expression stability of the candidate internal reference genes. However, accurate RT-qPCR analysis results cannot be obtained with only one single internal reference gene. Therefore, the geNorm is often used to determine the optimal number of reference genes under abiotic stresses and in different tissues. This can determine the most appropriate combination of internal reference genes for different experimental samples.

The *DREB* is a transcription factor unique to plants. Under adversity stresses, the *DREB* interacts with the DRE/CRT (dehydration response element) cis-element in the promoter region of the stress resistance genes, regulating the expression of a series of downstream genes (including DRE/CRT elements), and enhancing the resistance of plants to stresses [40,41]. The *DREB* gene can be induced to up-regulate its expression under the adversity stresses in *Musa acuminata* [42], *Glycine max* [43], and *Ricinus communis* [44]. The *POD* gene is a functional gene of antioxidant enzymes, and up-regulating its expression can help plants resist external damages when they encounter abiotic stresses [45]. This has been verified in *Phytophtora capsici* [46], *Ipomoea batatas* [47], *Tamarix hispida* [48], etc. Therefore, the *DREB* and *POD* genes can be used to verify the reliability of the screened reference genes. This study screened the most stable internal reference genes and their combinations under drought stress, salt stress, and heavy metal stress. Furthermore, the expression patterns of the *DREB* and *POD* genes in *B. papyrifera* under abiotic stresses were analyzed with the least stable genes as reference genes. When normalizing the gene expression levels with genes of stable expression, the expression patterns of the stress-responsive genes *DREB* and *POD* were consistent. However, when the unstable gene was used as a reference gene, the *DREB* and *POD* gene expression levels were significantly different. This further verified the accuracy of the screened internal reference genes. In summary, the selection of suitable internal reference genes is the key to analyzing the expression changes of target genes.

## 4. Materials and Methods

### 4.1. Materials

The seeds of *B. papyrifera* were collected from Yanji Town, Shuyang County, Suqian City, Jiangsu Province (N34.16560, E118.58537) in China. The seeds were soaked in 1600 mg L^−1^ Gibberellin A3 (GA3) solution (Coolaber, Beijing, China) for 24 h, rinsed with distilled water 2–3 times, and then sowed in a mixed substrate of peat soil (Pindstrup Mosebrug A/S, Ryomgaard, Denmark) and vermiculite (Guangdong Chenxing Agriculture Co., Ltd., Guangzhou, China). Then, they were cultured in a light incubator (LHP-300H, Changzhou Putian Instrument Manufacturing Co., Ltd., Changzhou, China) (at a temperature of 30 °C, with a humidity between 60% and 70%, a light intensity of 800 μmol m^−2^ s^−1^, and a photoperiod of 12 h light/12 h dark). After six months of culture, samples were collected from seven different tissues (i.e., terminal bud, young leaf, petiole, old leaf, phloem, xylem, and root). We selected seedlings of *B. papyrifera* with good growth and uniform size, carefully peeled off the nutrient matrix, placed them in the 1/2 Hoagland nutrient solution (Phygene Biotechnology Co., Ltd., Fuzhou, China) for one week, and then applied the abiotic stresses on the seedlings. The drought stress was simulated by a 30 g/L PEG-6000 solution (Tianjin Kermel Chemical Reagent Co., Ltd., Tianjin, China). The salt stress was carried out with a 300 mmol L^−1^ NaCl solution (Tianjin Kermel Chemical Reagent Co., Ltd., Tianjin, China). The heavy metal stress was applied by a 500 μmol L^−1^ ZnSO_4_·7H_2_O solution (Tianjin Bodi Chemicals Co., Ltd., Tianjin, China). Then, at 0 h (CK), 6 h, 12 h, 24 h, 48 h, and 72 h after the treatment, the roots, stems, and leaves were cut off and used as samples. Finally, the samples were frozen in liquid nitrogen immediately, and stored at −80 °C in an ultra-low temperature freezer (DW-HL668, Zhongke Meiling Cryogenics Co., Ltd., Hefei, China) until use. All the experiments were repeated three times.

### 4.2. Extraction and Detection of RNA

The RNA prep Pure Polysaccharide Polyphenol Plant Total RNA Extraction Kit (Cat. #DP441, Tiangen Biotech (Beijing) Co., Ltd., Beijing, China) was used to extract RNA according to the manufacturer’s instructions. In addition, 1% agarose gel (Biosharp Life Sciences, Anhui, China) electrophoresis was used to test the integrity of the RNA. Then, the concentration and purity of RNA were tested using an ultra-micro ultraviolet-visible spectrophotometer (NanoDrop2000) (NanoDrop2000, Thermo Fisher Scientific, Waltham, MA, USA).

### 4.3. Synthesis of cDNA

The RNase-free water was used to dilute the RNA to a concentration of 200 ng/μL, and the concentrations of the RNA were the same for all samples. To achieve a higher efficiency of synthesis, the RNA templates were incubated at 65 °C for 5 min, and then the samples were placed on ice for 2 min. According to the instructions of the M5 Super qPCR RT Kit (Cat. #MF012, Mei5 Biotechnology Co., Ltd., Beijing, China), the PCR reaction system was configured as follows: 4 μL 5 × M5 RT Super Mix and 2 μg RNA template were blended to a total volume of 20 µL with RNase-free water (Cat. #CD4381, Phygene, Biotechnology Co., Ltd., Fuzhou, China). The operations were performed on ice. Samples were reverse-transcribed using a gradient PCR amplification instrument from Bio-Rad (T100^TM^ Thermal Cycler, Bio-Rad, Hercules, CA, USA). The PCR reaction process was as follows: first incubated at 37 °C for 15 min, then incubated at 50 °C for 5 min, and finally heated at 96 °C for 5 min to deactivate the enzyme. After the reaction, the reverse-transcribed cDNA was stored at −20 °C for subsequent experiments.

### 4.4. Screening of Candidate Internal Reference Genes and Designing of Primers

According to the common internal reference genes in other plants in the existing literature, 15 candidate internal reference genes were selected according to the transcriptome database of *B. papyrifera*, namely: *NADH*, *L13*, *EIF3*, *HIS*, *Actin*, *PP2A*, *DOUB*, *UBE2*, *UBC*, *PTB*, *rRNA*, *GAPDH*, *HSP*, *RPL8*, and *TUA*. Primer 3web (http://primer3.ut.ee/, accessed on 1 June 2022) was used to design primers. The principles of primer design included: the length of the PCR amplification product between 100 bp and 300 bp, the primer length between 18 bp and 25 bp, the annealing temperatures between 50 °C and 60 °C, and the GC base content between 45% and 55%. It is necessary to avoid the occurrence of hairpin structures and primer-dimer mismatches as much as possible. In addition, the NCBI Primer-BLAST (https://www.ncbi.nlm.nih.gov/tools/primer-blast/index.cgi?LINK_LOC=BlastHome, accessed on 1 June 2022) was utilized to test the primer specificity. Primers were synthesized by General Biology (Anhui) Co., Ltd., Chuzhou, China.

### 4.5. RT-qPCR Reaction Conditions

The RT-qPCR used the SYBR green dye method, and the following PCR reaction system was created according to the instructions of the 2 × M5 HiPer SYBR Premix EsTaq kit (Cat. #MF787, Mei5 Biotechnology Co., Ltd., Beijing, China): 1 μL cDNA template, 0.2 μL both forward and reverse primers (10 μmol L^−1^), 3.6 μL ddH_2_O and 5 μL 2 × M5 HiPer SYBR Premix EsTaq. These operations were performed three times for all samples. The samples were amplified using the CFX96 RT-qPCR instrument from Bio-Rad (CFX96 Real-time System, Bio-Rad, Hercules, CA, USA). The PCR reaction programs consisted of pre-denaturation at 95 °C for 30 s, denaturation at 95 °C for 5 s, then annealing at 60 °C for 30 s, for 39 cycles (the melting curve was from 65 °C to 95 °C, increasing by 0.5 °C for each cycle, and lasting for 0.05 s to reach the melting temperature). The fluorescence signals were collected.

### 4.6. Detection of Primer Specificity and Amplification Efficiency

The cDNA samples were mixed in equal amounts and diluted three times with ddH_2_O as a template for the ordinary PCR amplification to test the specificity of the primers. Normal PCR amplification was performed according to the TaKaRa Taq (Cat. #R001A, TaKaRa, Kyoto, Japan) kit. The reaction system comprised 14.3 μL ddH_2_O, 2 μL 10 × PCR Buffer (Mg^2+^ plus), 0.1 μL TaKaRa Taq (5 U μL^−1^), 1.6 μL dNTP Mixture (2.5 mmol L^−1^), 1.5 μL cDNA, 0.25 μL upstream primers, and 0.25 μL downstream primers (10 μmol L^−1^). The reaction procedures consisted of 95 °C for 2 min; 98 °C for 10 s, 60 °C for 30 s, 72 °C for 30 s, 30 cycles, and 72 °C for 5 min at the end. After the reaction, the primer specificity was tested with a 1% agarose gel. The cDNA of all the samples were mixed in appropriate amounts and diluted into six gradients (1/3, 1/9, 1/27, 1/81, 1/243, and 1/729). These were then used as the templates to perform the RT-qPCR amplifications for a standard curve. In addition, the primer amplification efficiency was calculated by the formula:

E% = (3^−1/slope^ − 1) × 100% (1)

### 4.7. The Stability of Candidate Internal Reference Genes

The *C*_t_ of the 15 candidate genes in *B. papyrifera* was obtained via the RT-qPCR. The original *C*_t_ values were sorted out by using the software Microsoft Excel 2016, and three programs (geNorm [24], NormFinder [25], BestKeeper [26]) and the online analysis tool RefFinder [27] were operated to comprehensively evaluate the expression stability of the 15 candidate internal reference genes. Finally, the best internal reference genes in *B. papyrifera* under the abiotic stresses and in the various tissues were screened out.

### 4.8. Verification of the Expression Stability of the Internal Reference Genes

The genes in response to adversity stresses, i.e., *DREB* and *POD,* were selected to verify the stability of the screened internal reference genes. The cDNAs of the leaves of *B. papyrifera* under the drought stress, the stems of *B. papyrifera* under the salt stress, and the roots of *B. papyrifera* under the heavy metal stress were used as templates. By the RT-qPCR technology, the best candidate internal reference genes and their combinations were used as normalization factors, and the unstable internal reference genes were used for comparisons. Then, the relative expressions of the *DREB* and *POD* genes of *B. papyrifera* under the abiotic stresses were analyzed using the 2^−ΔΔCT^ method. The experiments were repeated three times. The reaction system and procedures were as described in Section 4.5.

## 5. Conclusions

In this study, 15 candidate internal reference genes were selected based on the transcriptome database of *B. papyrifera*, and their expression levels under abiotic stresses and in seven different tissues were studied. We used the programs geNorm, NormFinder, BestKeeper, and RefFinder to evaluate the expression stability of the candidate internal reference genes. Then, the accuracy of the screened reference genes was verified by the stress-responsive genes *DREB* and *POD*. This study provides a few reliable internal reference genes for the analysis of target gene expression in *B. papyrifera* under abiotic stresses and in the different tissues. This research lays the foundation for the study of the stress resistance and regulatory mechanisms in *B. papyrifera,* and the discovery of its important functional genes.

## Figures and Tables

**Table 1:** Primer sequence and amplification efficiency of the 15 candidate reference genes.

Gene	Primer ID	Primer sequence (5′-3′)	Amplicon Size (Bp)	Efficiency (%)	R^2^
*NADH*	*NADH*-F	GGACAGGTGGAAGATCGTCTG	111	97.18	0.987
	*NADH*-R	GGAATCTTCAGAACCCCGGAA			
*L13*	*L13*-F	TGCCAGCCCTAACTTTCATGT	126	92.17	0.999
	*L13*-R	AGACCCGGAGAAGAATTGCTC			
*EIF3*	*EIF3*-F	GTCCACATCATTCGAAGCAGC	130	106.31	0.997
	*EIF3*-R	GATCTATGAAGTGCCTGCGGA			
*HIS*	*HIS*-F	TGGCCTTGCATTCTCCAGTAG	118	98.87	0.996
	*HIS*-R	GACAAGCTGCGAGAGTGGTAT			
*Actin*	*Actin*-F	TACGCATTGAAGACCCTCCAC	148	90.26	0.998
	*Actin*-R	TGGCCACACTTGCTTAGACAA			
*PP2A*	*PP2A*-F	TCCTTTTGCGAGTCGATGGAA	119	117.99	0.988
	*PP2A*-R	CTTTGACGTTTGAAGCGAGCA			
*DOUB*	*DOUB*-F	CCTGATCTTCGCCGGAAAACA	194	97.48	0.999
	*DOUB*-R	TGGAGAGGGTTGAAGAGAGCT			
*UBE2*	*UBE2*-F	TCTCTGCTTACGGACCCAAAC	144	92.29	0.998
	*UBE2*-R	GAGGAGGAGCTATTGGGCCTA			
*UBC*	*UBC*-F	AGCATTACTTTCCGCTCCACA	119	91.44	0.995
	*UBC*-R	TGGCGAAAGTTTCTGTCCAGT			
*PTB*	*PTB*-F	CTGGAAACCTGCTGCCTTTTC	151	96.29	0.999
	*PTB*-R	ATTGAGGGTGTAGAAGCTGGC			
*rRNA*	*rRNA*-F	CAGGTTTCGATGTTGGGGAGA	196	95.56	0.999
	*rRNA*-R	CCAGCTTCCGAGAACATTCCT			
*GAPDH*	*GAPDH*-F	CCATGGAAGGACTTGGGGATC	156	90.43	0.995
	*GAPDH*-R	GTTCACTCCCACCACGTATGT			
*HSP*	*HSP*-F	CCAGCGCTGATGTTAGATTGC	174	92.66	0.993
	*HSP*-R	TTGCCATCAGAGCCTTTTCCT			
*RPL8*	*RPL8*-F	TGATCACCGACATCATCCACG	185	90.55	0.992
	*RPL8*-R	TCTGATCGGAAGGACATTGCC			
*TUA*	*TUA*-F	TCGAAAGGCCAACATACACCA	175	96.59	0.997
	*TUA*-R	GAGATGACAGGGGCATACGAG			
*POD*	*POD*-F	CTCCTGTGACCTCAACTGCAA	136	91.71	0.987
	*POD*-R	GAGTTGAACCATGGCGCAAAT			
*DREB*	*DREB*-F	TAAACCAGCTCACCCAATCCC	274	90.99	0.989
	*DREB*-R	CGGTTCTTGGGGAGTCTGATC			

**Table 2:** Expression stability of the reference genes in *B. papyrifera* calculated by NormFinder.

Rank	Drought Stress		Salt Stress		Heavy Metal Stress		Different Tissues		All Samples	
	Gene	Stability	Gene	Stability	Gene	Stability	Gene	Stability	Gene	Stability
1	*DOUB*	0.152	*DOUB*	0.172	*HSP*	0.359	*rRNA*	0.051	*rRNA*	0.338
2	*rRNA*	0.162	*HSP*	0.396	*rRNA*	0.362	*Actin*	0.222	*HSP*	0.383
3	*Actin*	0.394	*NADH*	0.540	*NADH*	0.401	*EIF3*	0.229	*NADH*	0.495
4	*HSP*	0.429	*rRNA*	0.569	*UBC*	0.513	*TUA*	0.343	*PP2A*	0.691
5	*UBC*	0.436	*PP2A*	0.630	*DOUB*	0.707	*DOUB*	0.346	*UBC*	0.738
6	*PTB*	0.520	*HIS*	0.635	*HIS*	0.745	*PP2A*	0.383	*Actin*	0.751
7	*EIF3*	0.547	*UBC*	0.654	*Actin*	0.805	*PTB*	0.383	*DOUB*	0.753
8	*TUA*	0.586	*L13*	0.720	*PP2A*	0.907	*HIS*	0.456	*PTB*	0.792
9	*PP2A*	0.596	*Actin*	0.742	*PTB*	0.969	*NADH*	0.484	*HIS*	0.966
10	*NADH*	0.616	*PTB*	0.817	*L13*	0.992	*HSP*	0.493	*L13*	1.028
11	*RPL8*	0.729	*TUA*	1.133	*RPL8*	1.265	*UBC*	0.503	*TUA*	1.090
12	*HIS*	0.917	*EIF3*	1.461	*TUA*	1.400	*L13*	0.557	*EIF3*	*1.277*
13	*L13*	0.998	*UBE2*	1.791	*GAPDH*	1.511	*RPL8*	0.836	*RPL8*	1.598
14	*UBE2*	1.090	*RPL8*	1.924	*EIF3*	1.672	*GAPDH*	1.178	*UBE2*	1.652
15	*GAPDH*	4.183	*GAPDH*	2.216	*UBE2*	1.921	*UBE2*	1.727	*GAPDH*	2.786

**Table 3:** Analysis of the expression stability by Bestkeeper and the ranking of the internal reference genes in *B. papyrifera*.

Rank	Drought Stress		Salt Stress		Heavy Metal Stress		Different Tissues		All Samples	
	Gene	Stability	Gene	Stability	Gene	Stability	Gene	Stability	Gene	Stability
1	*UBC*	0.398	*HSP*	0.453	*UBC*	0.382	*HSP*	0.215	*HSP*	0.518
2	*rRNA*	0.465	*NADH*	0.49	*NADH*	0.416	*RPL8*	0.216	*UBC*	0.535
3	*HSP*	0.481	*DOUB*	0.521	*HSP*	0.439	*DOUB*	0.256	*NADH*	0.607
4	*DOUB*	0.513	*UBC*	0.561	*rRNA*	0.493	*UBC*	0.475	*DOUB*	0.615
5	*PP2A*	0.515	*HIS*	0.596	*DOUB*	0.524	*NADH*	0.489	*Actin*	0.643
6	*Actin*	0.530	*PP2A*	0.609	*Actin*	0.607	*Actin*	0.514	*PP2A*	0.647
7	*EIF3*	0.557	*rRNA*	0.723	*PP2A*	0.645	*PP2A*	0.566	*rRNA*	0.672
8	*PTB*	0.634	*L13*	0.729	*HIS*	0.741	*L13*	0.588	*TUA*	0.886
9	*RPL8*	0.639	*Actin*	0.772	*L13*	0.768	*EIF3*	0.622	*L13*	0.913
10	*TUA*	0.671	*TUA*	0.784	*PTB*	0.866	*TUA*	0.641	*PTB*	0.917
11	*NADH*	0.727	*PTB*	0.871	*RPL8*	0.972	*rRNA*	0.650	*HIS*	1.018
12	*UBE2*	0.844	*EIF3*	1.336	*TUA*	1.088	*HIS*	0.651	*EIF3*	*1.023*
13	*L13*	0.928	*UBE2*	1.356	*GAPDH*	1.137	*PTB*	0.819	*RPL8*	1.353
14	*HIS*	0.982	*RPL8*	1.551	*EIF3*	1.303	*GAPDH*	1.213	*UBE2*	1.477
15	*GAPDH*	3.496	*GAPDH*	1.554	*UBE2*	1.593	*UBE2*	1.619	*GAPDH*	2.123

**Table 4:** Ranking of the expression stability of the reference genes in *B. papyrifera* by RefFinder.

Rank	Drought Stress		Salt Stress		Heavy Metal Stress		Different Tissues		All Samples	
	Gene	Stability	Gene	Stability	Gene	Stability	Gene	Stability	Gene	Stability
1	*rRNA*	2.115	*DOUB*	1.316	*HSP*	1.316	*EIF3*	2.28	*HSP*	1.414
2	*Actin*	2.449	*HSP*	1.414	*NADH*	2.06	*Actin*	3.13	*rRNA*	1.627
3	*UBC*	2.590	*NADH*	3.31	*rRNA*	2.632	*PP2A*	3.742	*NADH*	3
4	*DOUB*	3.130	*rRNA*	3.984	*UBC*	2.828	*rRNA*	4.031	*UBC*	3.761
5	*EIF3*	3.956	*HIS*	4.949	*DOUB*	5.477	*DOUB*	4.787	*PP2A*	5.091
6	*HSP*	4.899	*PP2A*	5.733	*HIS*	5.886	*HSP*	5.477	*Actin*	5.477
7	*PP2A*	6.160	*UBC*	6.293	*Actin*	6.735	*NADH*	6.344	*DOUB*	5.856
8	*PTB*	7.416	*L13*	7.737	*PP2A*	7.737	*UBC*	6.477	*PTB*	8.459
9	*NADH*	9.124	*Actin*	9	*PTB*	9.487	*L13*	7.502	*L13*	9.24
10	*TUA*	9.685	*PTB*	10.241	*L13*	9.487	*TUA*	7.933	*HIS*	9.975
11	*RPL8*	10.215	*TUA*	10.741	*RPL8*	11	*RPL8*	8.142	*TUA*	10.158
12	*HIS*	12.471	*EIF3*	12	*TUA*	12.243	*PTB*	8.596	*EIF3*	12
13	*L13*	13	*UBE2*	13	*GAPDH*	13.243	*HIS*	10.843	*RPL8*	13
14	*UBE2*	13.471	*RPL8*	14	*EIF3*	13.741	*GAPDH*	14	*UBE2*	14
15	*GAPDH*	15	*GAPDH*	15	*UBE2*	15	*UBE2*	15	*GAPDH*	15

## Data Availability

Not applicable.
